# The NK1 antagonist L-733,060 facilitates sequence learning

**DOI:** 10.1177/02698811231161582

**Published:** 2023-03-29

**Authors:** Natalia Favila, Kevin Gurney, Paul G. Overton

**Affiliations:** 1 Department of Psychology, The University of Sheffield, Sheffield, UK; 2 German Center for Neurodegenerative Diseases (DZNE), Bonn, NRW, Germany

**Keywords:** Action sequences, substance P, reinforcement learning, reward prediction error, striosomes

## Abstract

**Background:**

Although several brain regions and electrophysiological patterns have been related to sequence learning, less attention has been paid to the role that different neuromodulators play.

**Aims:**

Here we sought to investigate the role of substance P (SP) in sequence learning in an operant conditioning preparation, supported by a reinforcement learning model.

**Methods:**

Two experiments were performed to test the effects of an NK1 receptor (at which SP primarily acts) antagonist on learning and performing action sequences. In experiment 1, rats were trained to perform an action sequence until stable performance was achieved, and then, in phase 2, they were switched to perform the reverse sequence. In experiment 2, rats were trained to perform an action sequence, and in phase 2, they continued to do the same sequence. In both experiments in the first 3 days of phase 2, rats were injected with an NK1 receptor antagonist (L-733,060, i.p.) or with vehicle. Additionally, we developed a reinforcement learning model which allowed the in silico replication of our experimental tasks.

**Results:**

We found that administering an NK1 receptor antagonist weakened the stable retention of a well-learned sequence, allowing the faster acquisition of a new sequence, without impairing the continued performance of a crystallized sequence. Using our reinforcement learning model, we suggest that SP could be acting through the state value learning rate, modulating the effects of the reward prediction error.

**Conclusions:**

Our results suggest that SP could be involved in the consolidation of a sequence representation through a modulatory effect on the reward prediction error.

## Introduction

Most behavioral patterns are assembled from sequences of actions performed with a specific spatio-temporal organization. As action sequences are learned, they tend to become chunked or integrated into behavioral units, rendering their performance automatic and rigid (Dezfouli and Balleine, 2012; Sakai et al., 2003; Savalia et al., 2016; Smith and Graybiel, 2016). This is associated with a reduction in the cognitive load, thus being able to chunk behaviors is a fundamental process in the automatization of behavioral patterns, possibly allowing a more efficient execution of complex action sequences (Dezfouli et al., 2014; Smith and Graybiel, 2016; Veksler et al., 2014).

Several brain regions and electrophysiological patterns have been related to action sequencing. An overall picture has emerged across several species and tasks, in which the cortex seems to function as a master sending sensory, motor, and planning information to the striatum (Dhawale et al., 2021; Grillner, 2006; Kawai et al., 2015), which itself is one of the main structures for the acquisition and performance of action sequences (Geddes et al., 2018; Jin and Costa, 2015; Martiros et al. 2018; Nakamura et al., 2017; Ölveczky et al., 2005; Penhune et al., 2012; Smith and Graybiel, 2013; Yin, 2010). Although much information has been gathered about specific roles of striatal subregions (Geddes et al., 2018; Yin, 2010; Yin and Knowlton, 2006), firing patterns (such as the characteristic striatal bracketing activity; Jin and Costa, 2010; Jog et al., 1999; Martiros et al., 2018) and dopamine-dependent plasticity (Jin and Costa, 2015; Nakamura et al., 2017), less attention has been paid to the role that different striatal neuromodulators could be playing in action sequence chunking.

Within the striatum, there exists a complex biochemical circuit. Substance P (SP) is a neuropeptide abundant in the striatum that has been shown to facilitate the response of striatal medium spiny neurons (MSNs) to cortical glutamatergic inputs through presynaptic NK1 receptors (Blomeley et al., 2009). Interestingly, computational modeling studies have suggested that these SP connections could be essential for the smooth implementation of action sequences (Buxton et al., 2017). Furthermore, several studies have found a modulatory effect of SP on striatal dopamine (Brimblecombe and Cragg, 2015; Gauchy et al., 1996; Gygi et al., 1993; Kraft et al., 2001; Tremblay et al., 1992), which is itself instrumental in motor learning.

At the behavioral level, SP has been linked to learning, memory, and attention processes (Hasenöhrl et al., 2000; Lénárd et al., 2017; Porter et al. 2015). Using NK1 receptor knock-outs and NK1 receptor antagonists, it has been found that mice with impaired NK1 receptors display a greater percentage of omissions (i.e., they fail to respond), perseverative responses, premature responses, and they take longer times to retrieve the reward in the five-choice serial reaction time task (Yan et al., 2010; Weir et al., 2014; Porter et al., 2015; Porter et al., 2016). Although these results clearly suggest that impairment of NK1 receptors leads to disrupted action selection in an unordered serial task, few studies have focused on the role of SP in ordered action sequences that need to be integrated as a unit. Nevertheless, it has been reported that NK1 receptor antagonism leads to the inhibition of stereotypical behaviors (Duffy et al., 2002) and to disruptions inside the highly fixed and ordered grooming chain displayed by rats (Favila et al., 2021). Therefore, we hypothesized that SP could play a significant role in the changes in cortico-striatal synapses and dopamine that have been proposed as fundamental for learning and performing action sequences (Jin and Costa, 2015; Nakamura et al., 2017).

The aim of the present study was to investigate the effects of an NK1 receptor (primarily targeted by SP; Rupniak and Kramer, 2002) antagonist on learning and performing action sequences. To do so, in a first empirical experiment, we determined the effect of an NK1 receptor antagonist in a sequential reversal learning task (i.e., when rats had to reverse a sequence they had learned). In a second experiment, we determined the effects of an NK1 receptor antagonist when rats continued to performed a well-learned action sequence after achieving stable performance. Then, we developed a reinforcement learning model reproducing sequence learning under control conditions (i.e., without an NK1 receptor antagonist present), which gave us an in silico replication of our experimental tasks and allowed us to test hypotheses about the role of SP in the reinforcement learning algorithm. Our results suggest that SP could play a role in the maintenance of a sequence representation through a modulatory effect on the dopaminergic reward prediction error.

## Materials and methods

### Subjects

Twenty-one female Lister Hooded rats (200–300 g) purchased from Charles River (Kent, UK) were used. They were housed two or three per cage and kept on a 12-h light/dark cycle with free access to water at all times. Their weights were maintained at around 90% of their free-feeding weight by feeding them approximately 1 h every day after each experimental session. During the weekend, rats were allowed to free feed. All procedures were performed under the Scientific (Animal Procedures) Act 1986, as updated in 2010; Directive 2010/63/EU, and in accordance with the ethical guidelines of The University of Sheffield.

### Apparatus

All behavioral training and testing were carried out in Skinner-type operant chambers. Each chamber had two retractable levers on the frontal panel, one on the left (L) and one on the right (R). Above each lever there was a light that could be turned on and off. A food magazine was located between the two levers and had an infrared photobeam to register head entries. A 45 mg grain pellet (MLab Rodent Tablet 5TUM, TestDiet, Sawbridgeworth, UK) was used as a reinforcer and Arduino Microprocessors equipped with SD cards were used to control the operant chambers and to record levers presses and head entries. Each chamber had a ventilation fan, and an external white noise generator was used to mask extraneous sounds during all sessions.

### Experimental design

Two experiments were performed to test the effects of an NK1 receptor antagonist on reversal learning and performance of action sequences. In the first experiment, rats (*n* = 11) were trained to perform a heterogeneous sequence of two lever presses, either left-right (LR) or right-left (RL), for at least 25 sessions and until they displayed stable performance for five consecutive sessions. In a second phase, the reinforced sequence was switched, either from LR→RL or from RL→LR. On the first 3 days of this second phase, half of the rats (*n* = 6) were injected via an intraperitoneal route with saline, and half (*n* = 5) with the NK1 receptor antagonist L-733,060 (Tocris Bioscience, Abingdon, UK) at a dose of 2 mg/ml/kg. The waiting time between the injection and the experiment was 20 min. Both the dose and the waiting time were selected based on a previous study in which we found effects on innate sequential patterns with such dose and waiting time (Favila et al., 2021). The time the rats spent in the operant box after the injection depended on how long they took to obtain 50 reinforcers, with a maximum of 30 min. The second phase lasted 20 sessions in total, and on the last session a devaluation test was performed.

The second experiment was designed to test the effect of an NK1 receptor antagonist on the stable performance of a crystallized action sequence. In the first phase, rats (*n* = 10) were trained to perform a heterogeneous sequence of two lever presses, either LR or RL, for at least 25 sessions and until they reached stable performance for five consecutive sessions. In the second phase, the same action sequence continued to be rewarded, but on the first 3 days, half of the rats were injected via an intraperitoneal route with saline (*n* = 5) and half with L-733,060 (*n* = 5) at a dose of 2 mg/ml/kg. The second phase lasted 11 sessions, and a devaluation test was performed on the last session. A summary of the experimental designs of both experiments is shown in Table 1.

**Table 1.** Experimental design.

	Phase 1: Training	Phase 2: Testing		Devaluation test
Experiment 1 Reversal learning	Sequence training approx. 30 sessions (*n* = 11)	Switch to a new sequence 19 sessions		20th session
		Saline (*n* = 6)	L-733,060 (*n* = 5)	
Experiment 2 Performance	Sequence training approx. 30 sessions (*n* = 10)	Stay with the same sequence 10 sessions		11th session
		Saline (*n* = 5)	L-733,060 (*n* = 5)	

Two experiments were performed to test the effects of the NK1 receptor antagonist L-733,060 on reversal learning of an action sequence (experiment 1) and on performance of a well-learned sequence (experiment 2).

To control for any effects due to differences in the operant chambers or levers, rats were pseudo-randomly allocated to the operant chamber used (box 1 or 2), the reinforced sequence (LR or RL), and the experimental group (saline or L-733,060). Allocation to box and sequence were restricted such that each experimental group had rats distributed between the two operant chambers and the two possible sequences.

### Task and behavioral analysis

Behavioral training took place from Monday to Friday, with one session per day at approximately the same time every day. A free-operant approach was used, in which the length of the trials was not set a priori; thus rats could make different numbers of responses until the correct sequence of two responses was executed and the reinforcer was delivered. Training consisted of the following phases:

#### Magazine training

Rats were given two sessions of magazine training to allow them to learn where the pellets were delivered. Each session lasted until 20 reinforcers were randomly given or 20 min had elapsed without the need of any response.

#### Single lever training

Rats were initially trained to press each lever separately. To do so, every time the lever was pressed, the light above it was turned on and a reinforcer was delivered. Rats were kept in this phase until they had obtained 50 reinforcers in a single session with each lever. Which lever was trained first was randomized. This was the only phase of training in which external stimuli were used to guide behavior.

#### Switching training

Rats were reinforced for switching between the left and right levers with no specific order. Both LR and RL sequences were reinforced until rats had obtained 50 reinforcers per session in three sessions.

#### Sequence training

Finally, rats were trained to perform a single heterogeneous two-action sequence, either LR or RL, which sequence was reinforced was randomly assigned to each rat. When the correct sequence was performed it was followed immediately by the delivery of the reinforcer. A trial ended after the collection of the reinforcer, thus responses made before the collection of the reward were considered part of the trial (as shown in some trials in Figure 2(f)). All training sessions lasted until 50 reinforcers were delivered or 30 min had elapsed, whichever happened first. In the first five sessions of sequence training, rats were allowed to check the magazine between lever presses, but for the rest of the sessions, rats had to perform the correct sequence uninterruptedly without checking the magazine in the middle of the sequence. Training lasted for at least 25 sessions, and until the following performance criteria were met for five consecutive sessions:

1. The proportion of perfect trials was above 0.40. A trial was considered to be perfect if only one left and one right lever press were performed in the correct order.
2. The average number of lever presses per trial was between 2 and 3. Our target sequences were of two actions, thus we wanted to give little room for error.
3. The time between the responses of the reinforced action sequence was below 2 s, to ensure that rats were not doing other behaviors in between lever presses.
4. We used the ratio between the distal and proximal responses (DPratio) to capture preference for one of the levers. The responses of an action sequence can be classified with reference to how close in time they are from the reinforcer delivery. For example, in the sequence left-right, the left lever press would be the first response, and thus distal with respect to the reinforcer delivery, and the right lever press would be the second response and thus proximal to the reinforcer delivery. Thus, the ratio was calculated as:

$$\mathrm{DPratio}=\;\frac{\mathrm{Distal}\;\mathrm{lever}\;\mathrm{presses}}{\mathrm{Proximal}\;\mathrm{lever}\;\mathrm{presses}}$$

A ratio <1 indicates a preference for the temporally close response to the reinforcer, whereas a ratio >1 indicates a preference for the temporally distal response. Our criterion was that the ratio had to be 1 ± 0.25 to make sure that rats were not clearly favoring one of the levers.

#### Outcome devaluation test

At the end of the experiments, we performed an outcome devaluation test in which rats were free-fed for 1 h before being placed in the operant chamber for a 5 min extinction test, in which both levers were available but unresponsive and no reinforcers were delivered. Devaluation tests were performed with no feedback of any type so that rats’ performance relied solely on what was learned during training. The hypothesis was that if the rats had chunked the sequence, both levers would be equally affected by the devaluation as they would be integrated as a unit; on the other hand, if the rats had not chunked the two actions as a unit, the proximal lever would be more sensitive to the devaluation treatment because it is closer in time to the reinforcer, and thus, its press rate should be more depressed (Ostlund et al., 2009).

### Statistical analysis

We performed two-factor mixed analyses of variance (ANOVAs) to analyze how the between variable Treatment (saline vs L-733,060) and the within variable Session (1, 2, 3, . . .) affected the following performance metrics: proportion of perfect trials, inter-response times, actions or presses per trial, presses per minute, and distal/proximal ratio. Given that rats could spend a variable number of sessions in the first phase depending on how fast they reached our criteria, and we were mainly interested in the rats reaching a similar stable performance, for the statistical analysis we only considered the first five and last five sessions of the first (pre-drug) phase of both experiments.

For the second phase of the first experiment, we observed that from session 1 to 8, learning occurred quickly; whereas, from session 9 onwards the changes in performance were much slower. Thus, we divided our analysis of the second phase into early (sessions 1–8) and late (sessions 9–19) learning as has been done previously (Nakamura et al., 2017). Furthermore, we performed one-way ANOVAs to compare the number of sessions rats spent learning the first action sequence, to ensure there were no systematic baseline learning differences between our control and experimental groups. Whenever an interaction was found significant, post hoc pairwise *t*-tests with Bonferroni corrections were performed. For all tests performed *p* *<* 0.05 (two tailed) was considered significant. Effect sizes were calculated using Cohen’s *d*. Results are presented as mean ± standard error of the mean. All statistical analyses were performed using software R studio (https://www.rstudio.com/).

## Reinforcement learning model

### Model description

**Figure 1.**
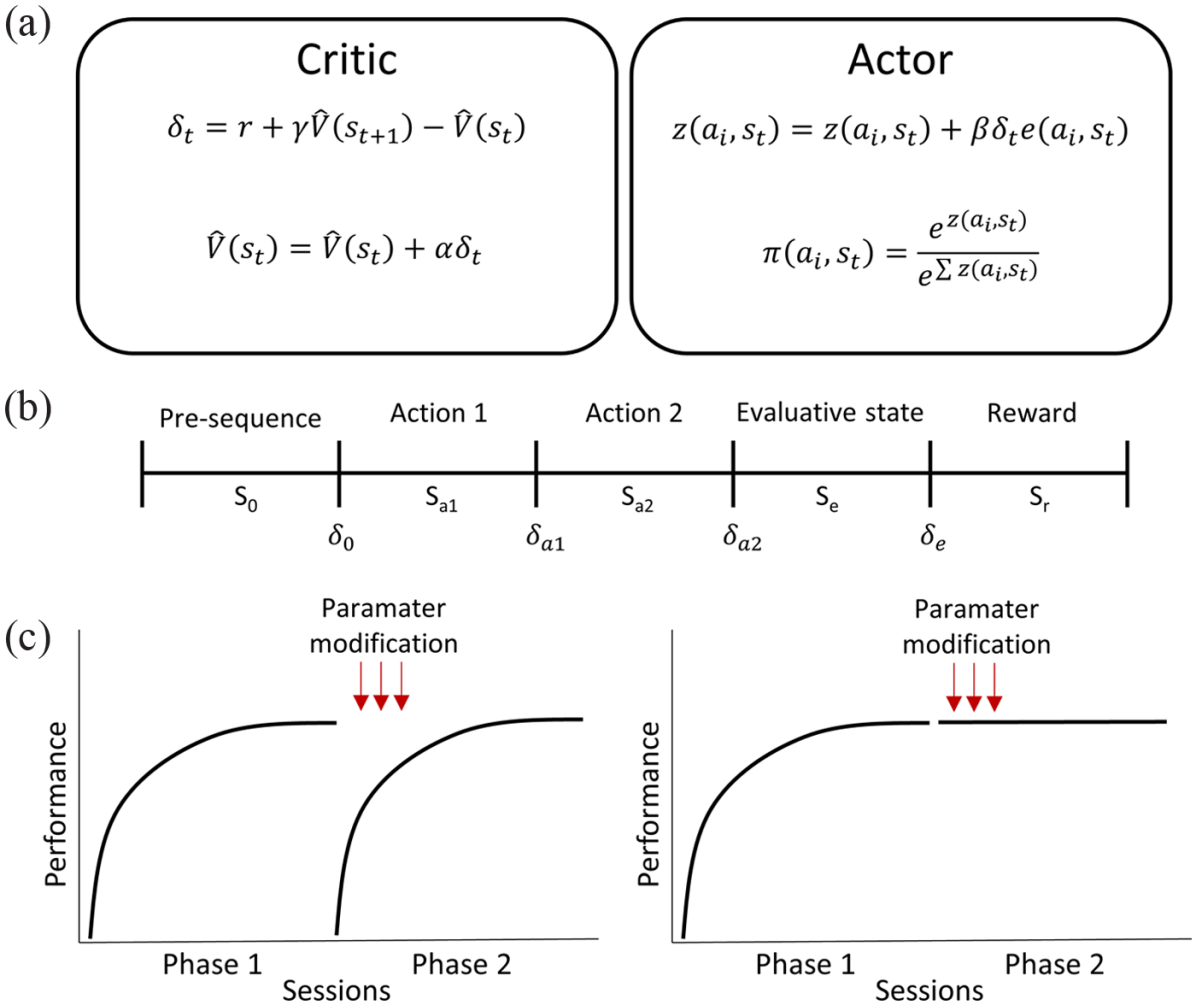
Model-free reinforcement learning algorithm for a two-action sequence task. (a) Summary of the model used with an actor critic paradigm as overarching architecture. The critic is in charge of updating the value of the states and calculating the reward prediction error, while the actor is in charge of updating action preferences and selecting actions based on a softmax selection policy (see main text for symbol definitions). (b) Division of the state space of the model into biologically significant states to learn a two-action sequence. (c) Simulations performed to replicate the two empirical experiments. In the first simulation agents were trained to perform the action sequence LR for 30 sessions until stable performance was achieved; in a second phase they were switched to do the reverse sequence RL. In the second simulation, agents were trained to perform the sequence LR until stable performance was achieved, and in the second phase they continued to perform the same sequence. In both simulations, different parameters were modified during the first 100 trials of the second phase to try to simulate the effects of the substance P antagonist injected in the experiments. LR: left-right; RL: right-left.

We developed a reinforcement learning model to test biologically constrained hypotheses about the role of SP in reinforcement-based sequence learning. We used temporal difference learning with an actor-critic paradigm as an overarching architecture (Joel et al., 2002; Sutton and Barto, 1998). A summary of our model is presented in Figure 1(a).

#### Learning

Reinforcement learning agents learn to estimate the value of actions and states through trial and error in order to maximize reward (Sutton and Barto, 1998). Thus, let $\hat{V}\left(s_{t}\right)$ be the estimated value of state $s_{t}$ at time *t*; $\hat{V}\left(s_{t+1}\right)$ the estimated value obtained in the next state $s_{t+1}$ reached after taking action $a_{i}$ at time *t*; r the reinforcer procured after taking action $a_{i}$ , and γ the discount factor, accounting for the fact that future states are temporally distant and thus less valued. Then, the reward prediction error (RPE), $\delta_{t}$ , is calculated as follows:

$$\delta_{t}=r+\gamma \hat{V}\left(s_{t+1}\right)-\hat{V}(s_{t})$$

This term is coding the difference between the expected value, $\hat{V}\left(s_{t}\right),$ and the discounted value of the state reached, $\gamma \hat{V}\left(s_{t+1}\right),$ plus the reward obtained, r . Thus, informally, $\delta_{t}$ tells the agent whether there was an improvement or not after taking action $a_{i}$ in state $s_{t}$ .

Let α<1 be a learning rate for updating state values, and β<1 the learning rate for updating a set of action preferences, $z\left(a_{i},s_{t}\right)$ , which are used to choose between actions according to the policy defined below. The RPE is used to update the estimated value of the states and action preferences in the following way:

$$\hat{V}\left(s_{t}\right)=\hat{V}\left(s_{t}\right)+\alpha \delta_{t}$$

$$z\left(a_{i},s_{t}\right)=z\left(a_{i},s_{t}\right)+\beta \delta_{t}$$

Thus, if the agent ended up obtaining a reward or in a better state than it was expecting, $\delta_{t}$ will be positive and the estimated value of the state and action previously taken will increase proportionally to α and β .

*Choice*. Action selection was performed via a policy of softmax selection using the action preferences. In this policy, the probability of an action is given by:

$$\pi \left(a_{i},s_{t}\right)=\frac{e^{z(a_{i},s_{t})}}{e^{\sum^{z}(a_{i},s_{t})}}$$

such that actions with higher values are more likely to be selected, but not in a deterministic way, so there is still a small probability that other actions will be picked, promoting exploration.

#### Credit assignment

To capture the credit assignment problem, in which rats initially assign credit to the proximal response performed right before the delivery of the reward rather than to the whole action sequence, we added eligibility traces to the action preferences (Sutton and Barto, 1998). Eligibility traces account for the fact that temporally distant actions from the reinforcer are less affected by the RPE than those closer to it.

To implement them, we added a memory variable, $e(a_{i},s_{t})$ , associated with each action-state pair. Let λ be a decay parameter, controlling how much previous actions are affected by the current RPE, and γ the discount factor previously mentioned. Then, at each time step, if an action is performed, its eligibility trace increased to 1 and the eligibility trace of the other action decayed by a factor of γλ . That is:

$$e\left(a_{i},s_{t}\right)=\;\{\begin{array}{c} \gamma \lambda e\left(a_{i},s_{t}\right)\;if\;a_{i}\;was\;not\;perfomed\\ 1\;if\;a_{i}\;was\;perfomed \end{array}$$

If λ=1 , all previously performed actions are remembered perfectly and all are given credit for the reward. If λ=0, then only the most recently performed action is given credit, and it is the only one affected by the RPE.

The addition of the memory variable $e(a_{i},s_{t})$ makes the update of the action preferences in the following way:

$$z\left(a_{i},s_{t}\right)=z\left(a_{i},s_{t}\right)+\;\alpha \delta_{t}e(a_{i},s_{t})$$

Thus, eligibility traces modulate which actions performed are eligible to undergo learning changes produced by RPE $\delta_{t}$ .

### Model space

Previous work has divided the state space of reinforcement learning models into a large number of equal time slots and states, to capture some of the continuity of time and space, for example, Schultz et al. (1997) divided their trials into 60 time-states. To capture the nature of the tasks of our learning experiments, we decided to perform the division of the states into what seemed biologically significant, according to the task our subjects performed when learning an action sequence (Figure 1(b)).

Thus, the simulations’ states were divided into (1) a pre-sequence state (S_0_), (2) a state for performing the first action (S_a1_), (3) a state for performing the second action (S_a2_), (4) an evaluative state (S_e_), and (5) a reward state (S_r_), as shown in Figure 1(b). Given that different actions can take the agent to different states, there were two separate evaluative and reward states, depending on the actions performed in the two previous states. If the correct action sequence had been performed in S_a1_ and S_a2_, then the agent moved toward an evaluative and reward state associated with a positive reward of one. If the agent selected any other combinations of two responses that was incorrect, it ended up in a no-reward state, and a small penalty of −0.05 was given, representing energy costs of performing incorrect actions. The logic behind this division was that the reinforcement program used in the real experiments was continuously evaluating the last two responses the rat had performed, and, if the correct sequence had been executed, it delivered a food pellet. On the other hand, if the last two responses were not the reinforced sequence, no-reward was given. Although it might take a while for the rats to actually get to this representation of the environment, it was not the main purpose of this study to formalize the development of the representation of the states per se.

### Parameter optimization

To select the parameters of the model, we optimized the model against the behavioral data of rats learning a sequence without the NK1 receptor antagonist injection, that is, using the learning data from the first pre-drug phase. Then, we used this model to test hypotheses about the step in the reinforcement learning model at which SP could be acting by modifying the model parameters and analyzing whether we could replicate the results when the NK1 receptor antagonist was injected. To do this, the initial values of the parameters of the model were picked based on minimizing the distance between the sequence learning data obtained from 21 rats and the data obtained from simulated agents learning a two-action sequence.

Given that in the experiments rats needed a variable number of sessions until displaying stable performance, to tune the parameters of the model we used 25 sessions of the rats’ data, consisting of their first 20 sessions and the last five sessions of training of each rat. For the simulated agents, we sampled the parameter space for the learning rates (α and β), the discount factor (γ), and the eligibility trace parameter (λ), in a range from 0 to 1 in steps of 0.1. Two performance measurements were calculated for each combination of parameters: proportion of perfect trials and distal/proximal ratio. The final parameter values were selected based on the minimum mean square error obtained from the difference between the rats’ learning data and the simulated agents’ data.

We ended up picking the parameters that minimized the difference between the real and simulated distal/proximal ratio measurement, given that this provided on average the smallest mean square error for both performance measurements and, although it gave a slightly higher error for the proportion of perfect trials, the shapes of the learning curves were similar. The final parameter values of the model are displayed in Table 2. Finally, the model of all simulated agents was initialized such that all state values and eligibility traces were set to zero and the action preferences were set to 5 to make sure that they were not biased toward any of the actions at the beginning of training.

**Table 2.** Parameter values of the reinforcement learning algorithm.

Parameter	Value
α	0.1
β	0.1
γ	0.9
λ	0.1

α: state learning rate; β: action learning rate; γ: discount factor; λ: eligibility trace decay.

### Replicating the experimental structure in simulations

We ran two groups of simulations to reproduce the structure of the experiments performed. Figure 1(c) shows an illustration of the structure of the simulations. In the first simulation, replicating our first learning experiment, simulated agents were trained to perform a two-action sequence for 30 sessions, and then, in a second phase, the learning contingency was reversed, such that the agents had to reverse the order of the actions to obtain the reward for another 30 sessions. In the second simulation, replicating our second experiment, simulated agents were trained to perform a two-action sequence for 30 sessions, and then, in the second phase they were kept performing the same sequence for another 20 sessions. During the first 100 trials of the second phase of both simulations, different parameters of the model were modified to try to simulate the effects produced by the NK1 receptor antagonist injection on the experimental animals’ performance. All sessions in both simulations lasted until 50 rewards were obtained, just as in the real experiment.

#### Code availability

The code for the reinforcement learning model is in Matlab and is freely available at https://github.com/NataliaFavila/RL_model_action_sequences.git.

#### Data availability

The behavioral datasets generated and analyzed during the current study are available from the corresponding authors upon request.

## Results

### NK1 receptor antagonist enhances performance in learning a new sequence

**Figure 2.**
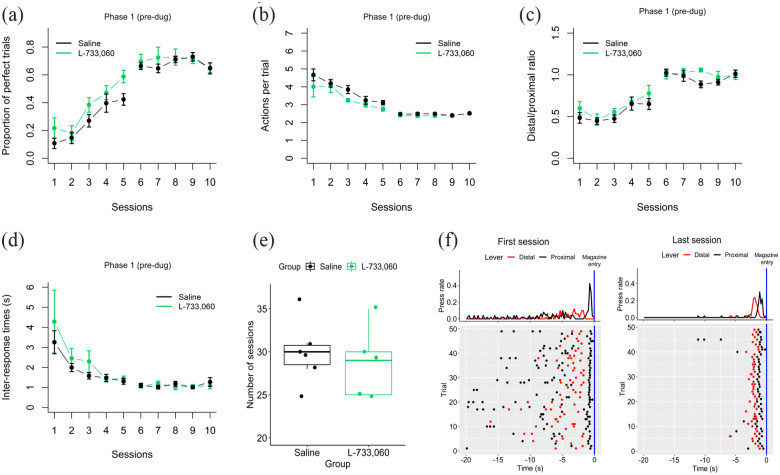
First phase (pre-drug) action sequence learning. Average performance of the control group (black line) and the L-733,060 group (green line) in the first (pre-drug) phase. All plots depict the first five and last five sessions of the first phase of the following performance metrics: (a) Proportion of perfect trials. (b) Mean number of actions performed per trial. (c) Distal/proximal ratio. (d) Mean inter-response times between the actions of the reinforced sequence. (e), Number of sessions needed to learn the first action sequence. (f) Trial by trial example of a rat learning to perform the sequence Left-Right in the first (left panel) and last (right panel) session of training. Behavior was aligned to the moment in which the rat collected the reward (blue line). Data are shown as mean ± standard error of the mean.

We began by checking that rats actually learned the trained sequence and that there were no significant differences between saline and L-733,060 animals in learning the first (pre-drug) sequence. A 2 × 10 mixed ANOVA performed on the first phase showed that there was a significant Session effect in all performance measurements, with a significant increase in the proportion of perfect trials (*F*(9,81) = 57.77; *p* = 2e-13; Figure 2(a)), a decrease in the mean number of actions performed per trial, approaching two actions (*F*(9,81) = 33.21; *p* = 2e-16, Figure 2(b)), a shift toward 1 in the distal/proximal ratio (*F*(9,81) = 46.11; *p* = 2.8e-09, Figure 2(c)), and a significant reduction in the mean time between responses of the reinforced sequence, reaching an average of 1 s (*F*(9,81) = 8.89; *p* = 8.6e-09; Figure 2(d)). However, there were no significant main effects of Treatment or Treatment × Session interactions in any of the performance measurements, nor a significant Treatment effect (*F*(1,9) = 0.26; *p* = 0.62; Figure 2(e)) in the number sessions needed to reach the performance criteria between the two groups, confirming that there were no differences in how both groups learned the first (pre-drug) action sequence.

Furthermore, our training regime led animals to perform the learned action sequences in a very precise pattern. Figure 2(f) shows an example of the behavior of a rat in the first and last session of training of the first phase (pre-drug). Each dot is a behavioral response either to the left (red) or right (black) lever, and each row represents one trial. Time zero is the moment when the rat put its head in the magazine to collect the reinforcer. In the first session (left panel Figure 2(f)) this rat performed many more responses than the two needed to obtain the reward. While in the last session (right panel Figure 2(f)), with the odd exception, the rat had crystallized its performance into a very stable spatio-temporal sequence, suggesting that our training program led to highly accurate performance.

**Figure 3.**
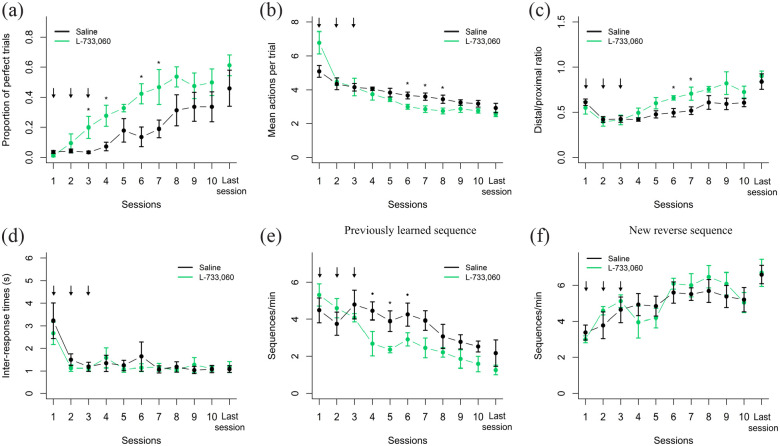
Effects of L-733,060 on learning a new sequence. Average performance of the saline and L-733,060 groups on the second phase of experiment 1, when the reverse sequence had to be learned. The performance metrics analyzed were (a) proportion of perfect trials; (b) mean actions per trial; (c) distal/proximal ratio; (d) inter-response times between the actions of the reinforced sequence; (e) press rate (sequences per min) at which rats stopped performing the first learned action sequence; (f) press rate at which rats performed the new reinforced sequence. Only the first eight sessions and the last session of the second phase are displayed. Data are shown as mean ± standard error of the mean. Black arrows indicate the sessions in which L-733,060 was administered. *p < 0.05, •*p* < 0.10.

Once the first sequence had been learned, rats were moved on to the second phase, in which to obtain the reward they now had to perform the reverse sequence, and during the first three sessions of this second phase they were injected with either L-733,060 or saline. Figure 3 shows the results from the first 10 days and the last session of this second phase. A 2 × 8 mixed ANOVA showed a significant main Treatment effect (*F*(1,9) = 9.46; *p* = 0.013), Session effect (*F*(7,63) = 13.21; *p* = 2.4e-10) and a marginally significant Treatment × Session interaction (*F*(7,63) = 2.15; *p* = 0.05) on the proportion of perfect trials (Figure 3(a)), suggesting a better performance for the rats injected with L-733,060. Post hoc *t*-tests indicated that the rats injected with L-733,060 actually learned the new sequence faster than saline rats, with significant differences in session 3 (*t*(9) = −2.50, *p* = 0.03, *d* = 1.51), session 4 (*t*(9) = −2.85, *p* = 0.01, *d* = 1.73), session 6 (*t*(9) = 3.06, *p* = 0.01, *d* = 1.85), and session 7 (*t*(9) = −2.27, *p* = 0.04, *d* = 1.37).

This faster increase in the proportion of perfect sequences when L-733,060 was injected was accompanied by a better performance in other performance measurements as well. There was a significant Treatment × Session interaction in the mean actions per trial (*F*(7,63) = 4.59; *p* = 0.0003, Figure 3(b)) and in the distal/proximal ratio (*F*(7,63) = 2.43; *p* = 0.03, Figure 3(c)). However, we did not find a significant differences in the time between responses (*F*(7,63) = 0.31; *p* = 0.59, Figure 3(d)) or in the total time rats took to complete each session (*F*(7,63) = 2.0; *p* = 0.07, Figure S1). Thus, the rats injected with L-733,060 learned the reverse sequence faster than control rats, but without effects on the speed at which they performed the responses or on how much time overall they spent on the task. The effect of L-733,060 seems to fade after session 8, since there were no significant Treatment or Treatment × Session interactions in the last 11 sessions in any of the behavioral metrics. The last data point of all plots of Figure 3, belonging to the last session of the second phase, shows that eventually both groups reached similar performance.

We then further analyzed whether the NK1 receptor antagonist differentially affected the extinction of the first sequence learned versus the learning of the new reverse sequence. A 2 × 8 mixed ANOVA showed a significant Session effect (*F*(7,63) = 4.53; *p* = 0.0004) and Treatment × Session interaction (*F*(7,63) = 2.35; *p* = 0.03) on the rate at which rats extinguished the first learned sequence (Figure 3(e)). Post hoc pairwise *t*-test revealed significant differences in session 5 (*t*(9) = 2.44, *p* = 0.04, *d* = 1.47), and marginally significant differences on sessions 4 (*t*(9) = 2.91, *p* = 0.05, *d* = 1.32) and 7 (*t*(9) = 1.96, *p* = 0.08, *d* = 1.19), suggesting that rats injected with L-733,060 stopped performing the previous sequence faster. On the other hand, the rate at which the new sequence was performed consistently increased, with a significant Session effect (*F*(7,63) = 11.47; *p* = 2.7e-09, Figure 3(f)); but with no significant Treatment effect (*F*(1,9) = 0.05; *p* = 0.83) or Treatment × Session interaction (*F*(7,63) = 1.45; *p* = 0.20), indicating that the effect of L-733,060 seems to be limited to extinguishing the first learned sequence.

**Figure 4.**
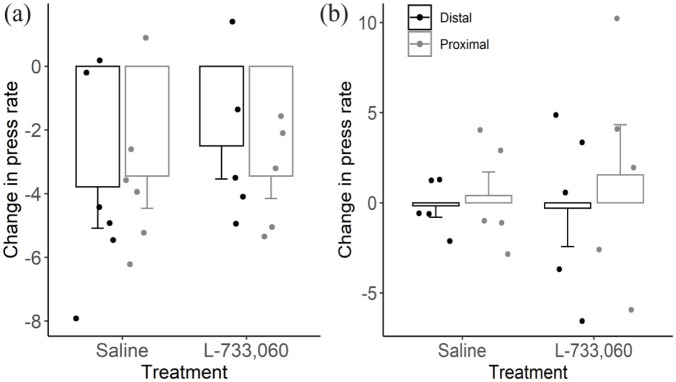
Devaluation tests. Change in presses per minute between the last session of training and the extinction test for the distal (white) and proximal (black) lever during the 5 min extinction test. (a) Results from the learning experiment. (b) Results from the performance experiment. Data are displayed as mean ± standard error of the mean.

A devaluation test was performed to assess whether rats from each group had similar representations of the second learned sequence at the end of the experiment. Figure 4(a) shows for the distal and proximal actions the change in press rate from the last session of training to the extinction session for the saline and L-733,060 rats. Both groups displayed a similarly decreased press rate in both levers during extinction with no significant Treatment (*F*(1,9) = 0.21; *p* = 0.65) or Lever effect (*F*(1,9) = 0.12; *p* = 0.73), suggesting that both groups performed similarly in the devaluation test. Overall, the devaluation test results suggest that the effects of the NK1 receptor antagonist were limited to the moment in which the contingencies were switched and to the extinction of the first learned sequence.

### NK1 receptor antagonist does not affect performance of well-learned action sequences

**Figure 5.**
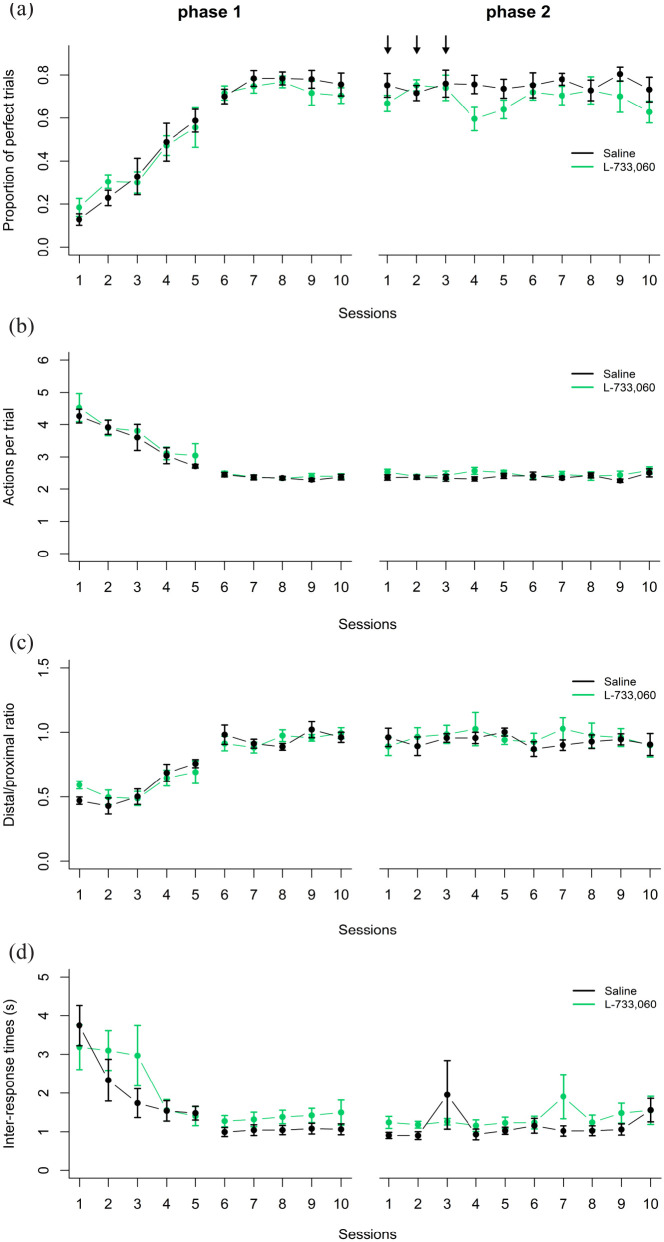
Effects of L-733,060 on sequence stable performance. The first phase (first and last five sessions) is shown on the left panel and the complete second phase on the right panel. L-733,060 was injected in the first three sessions of the second phase. Performance metrics analyzed were (a) proportion of perfect trials; (b) mean actions per trial; (c) distal/proximal ratio; (d) inter-response times between the actions of the reinforced sequence. Data are shown as mean ± standard error of the mean. Black arrows indicate the sessions in which L-733,060 was administered. *p < 0.05. •*p* < 0.10.

The second experiment was performed to test the effect of an NK1 receptor antagonist on the stable performance of a well-learned sequence. We first trained two groups of rats to perform a two-action sequence until they had stabilized their performance according to our criteria. There was no significant difference in the number of sessions needed to learn this first (pre-drug) sequence (*F*(1,8) = 0.02, *p* = 0.87), with both groups of rats reaching stable performance in 29 sessions in average. Furthermore, we found a significant Session effect on the proportion of perfect trials (*F*(9,72) = 50.21, *p* = 1.2e-11, Figure 5(a), left panel), the actions performed per trial (*F*(9,72) = 32.71, *p* = 2.7e-14, Figure 5(b) left panel), the distal/proximal ratio (*F*(9,72) = 34.60, *p* = 4.8e-08, Figure 5(c) left panel), and on the inter-response times (*F*(9,72) = 17.13, *p* = 1.8e-07, Figure 5(d) left panel), suggesting that as sessions progressed rats were able to correctly learn to perform the reinforced sequence. However, no significant Treatment or Treatment × Session interaction effects were found in any of the performance measures during the first phase, indicating that all rats from the control and the L-733,060 group learned the (pre-drug) sequence in a similar fashion.

Once rats had learned the action sequence, they were moved to the second phase, in which the same sequence was reinforced, but rats were injected with either saline or the L-733,060 (2 mg/kg) for three consecutive days. Results for this second phase are shown on the right panels of Figure 5(a), (b), (c) and (d). No significant Session, Treatment, or Treatment × Session interaction effects were found in the proportion of perfect trials (*F*(9,72) = 1.75, *p* = 0.09, n. s.), actions per trial (*F*(9,72) = 1.32, *p* = 0.24, n. s.), distal/proximal ratio (*F*(9,72) = 0.61, *p* = 0.78, n. s.), or inter-response times (*F*(9,72) = 1.18, *p* = 0.31, n. s.). There was also no effect on the rate at which the reinforced sequence was performed (*F*(9,72) = 1.33, *p* = 0.23, n. s.), or in the other possible sequence (*F*(9,72) = 0.53, *p* = 0.84, n. s.), indicating that injecting L-733,060 had no effect on the stable performance of the learned action sequence.

At the end of the second phase, we performed a devaluation test. There was no significant Treatment effect (*F*(1,8) = 0.04, *p* = 0.85) or Lever effects (*F*(1,8) = 2.66, *p* = 0.14, Figure 4(b)) on the change in press rate, suggesting that by the end of the experiment both groups had similar representations of the learned sequence. Furthermore, in contrast to the first experiment, the changes in press rate were closer to zero for both levers, indicating that in the second experiment rats kept pressing both levers under extinction conditions at a similar rate as in the last session of training, even though no reinforcers were given. In this second experiment rats were trained in the same sequence for a longer time, thus, it makes sense that their press rates were less sensitive to the devaluation of the reinforcer.

### Computational modeling: Validating the model

**Figure 6.**
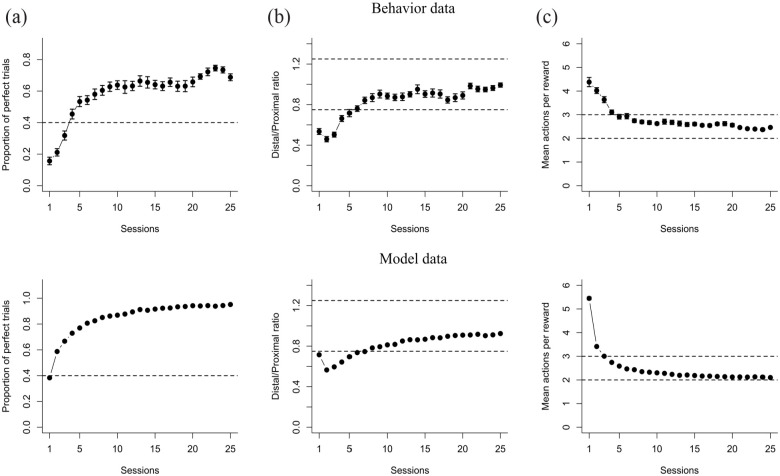
Validation of the reinforcement learning algorithm versus experimental data. Performance of the simulated agents (bottom plots) and the experimental data (top plots) in the following performance metrics: (a) Proportion of perfect trials, (b) distal proximal ratio, and (c) Mean actions per trial. Experimental data (top plots) show the mean performance of the first 20 and the last five sessions of each rat. Data are shown as mean ± standard error of the mean.

We first validated that our reinforcement learning agents learned a two-action sequence in a similar way to the experimental rats. Just as in the experiments described earlier, our simulated agents were only reinforced when the correct actions were performed in the correct order in session that lasted until 50 reinforcers were obtained. Figure 6 shows the mean proportion of perfect trials (Figure 6(a)), mean distal/proximal ratio (Figure 6(b)), and the mean actions per trial (Figure 6(a)) for 21 rats in the top panels and for 100 simulated agents in the bottom panel. Figure 6(a) shows that similarly to rats (top plot), the simulated agents (bottom plot) gradually increased the proportion of perfect trials, until reaching stable performance. Furthermore, in Figure 6(b), bottom plot, we show that simulated agents displayed a strong credit assignment error, shown at the beginning of training by a distal/proximal ratio below one, which indicates a preference for the proximal action. However, as sessions progressed, agents were able to perform both actions at a similar level, indicated by a distal/proximal ratio that gradually approached a value of one. Furthermore, in a similar way to rats, our simulated agents gradually decreased the mean number of actions until approaching two actions, which was the length of the target sequence (Figure 6(c)). Finally, it is worth noting that the model was trained in approximately the same number of sessions as the rats, with rats needing between 25 and 45 sessions to achieve our performance criteria, and the model around 25 sessions to reach stable performance.

In summary, as learning occurred, both simulated and experimental data showed a gradual increase in proportion of perfect trials, a refinement in the number of actions performed to obtain the reward, and a credit assignment problem that gradually dissipated as learning was crystallized, suggesting that our reinforcement learning model was able to replicate, in general terms, the basic behavioral phenomena observed when rats learn a two-action sequence. However, it is worth noting that although we look for a combination of parameters that led to the smallest root mean square error in the performance measurements, the reinforcement learning agents did eventually get to a better performance level than the experimental rats, performing almost all trials perfectly. Such small discrepancies are to be expected since it is not possible to capture every variable acting on the performance of the rats. Nevertheless, the shape of the three performance measurements used here display the same basic trends of action sequence acquisition.

### SP modulation of the reward prediction error through the state learning rate

We then used the developed model to test hypotheses about SP’s role in sequence learning. It is well known that dopamine is a very important neurotransmitter for learning new behaviors, and Brimblecombe and Cragg (2015) have reported that SP modulates dopamine release in the striatum’s striosomes but not on the matrix. Interestingly, given their input-output structure, striatal striosomes have been suggested to encode state values (Amemori et al., 2011; Doya, 2002). Thus, we hypothesized that the injection of the NK1 receptor antagonist in our experiments might have downregulated the learning rate of the state values, α, which modulates the effect of the RPE, believed to be encoded by dopamine (Schultz et al., 1997).

**Figure 7.**
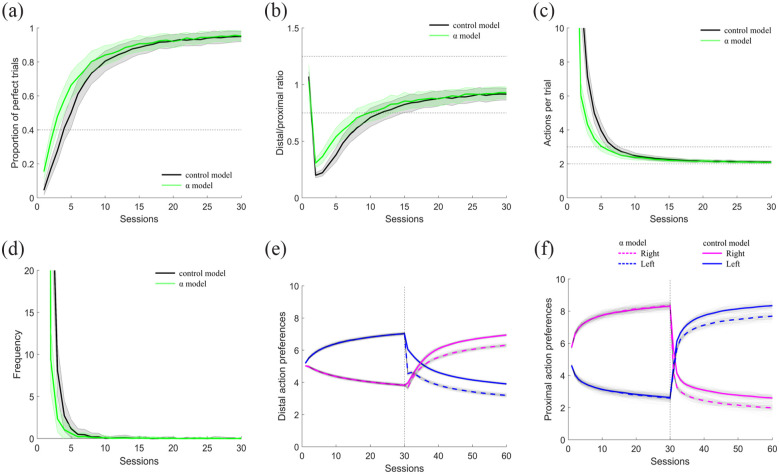
Effects of modifying the state learning rate α on learning a new sequence. Results are only for the second phase of the simulation of experiment 1. In the first 100 trials of this second phase α was reduced for the α model (green line) and kept unaltered for the control model (black line). Results show performance of the two models in the: (a) Proportion of perfect trials, (b) distal/proximal ratio, (c) mean actions per trial, and (d) extinction of the first learned sequence. (e) and (f) Changes in the action preference in the distal and proximal positions throughout the two phases of the simulation of experiment 1 for the α model (dotted lines) and the control models (continuous line). Data are shown as mean ± standard error of the mean.

To simulate the learning experiment, we first trained two groups of 100 agents to perform a two-action sequence (LR). This first sequence was learned similarly in both groups of agents (Fig. S2, Supp Mat). Then, in a second phase we flipped the reinforced sequence, from LR→RL, and during the first 100 trials of the second phase we decreased parameter α from 0.1 to 0.03 for one of the groups of simulated agents, we called this the α model, while the model with no parameter modification is called the control model. Figure 7 shows the results of this manipulation on the second phase of the learning simulation, that is, when agents had to forget the first learned sequence and learn a new one. This simple manipulation was able to replicate the trends of our first experiment (see Figure 3(a), (b) and (c)). Similar to our rats injected with an NK1 receptor antagonist, our simulated agents with decreased α learned the reverse sequence faster, with a faster increase in the proportion of perfect trials (Figure 7(a)), a faster dissipation of the credit assignment problem (Figure 7(b)) and a faster approach to the target length of the sequence (Figure 7(c)). Interestingly, just as with the rats (Figure 3(e)), the agents with a reduced α extinguished the first learned sequence faster than the control agents (Figure 7(d)).

To understand why the simulations learned the new sequence faster, we plotted the action preferences when the distal and proximal actions were performed to see how they changed through both phases of the simulation (Figure 7(e) and (f)). We can see that all agents learn to increase their preference for the correct distal and proximal action in the first phase. This led to very high probabilities of doing left action (blue line) in the distal position (Figure 7(e)) and the right action (magenta line) in the proximal position in phase 1 (Figure 7(f)), following a similar learning curve as the proportion of perfect trials (Figure 7(a)). In the second phase, that is when agents had to reverse the learned action preferences in order to obtain the reward, we can see that while the control model (solid lines) showed a gradual flip of the action preferences, the downregulation of α produced a faster decay of the previously learned action preferences (dashed lines) at the beginning of the second phase. This is particularly visible in the distal position (Figure 7(e) and (f), signaled by an arrow). This decay made the preferences of both actions more similar to each other at the beginning of the second phase, which had the effect of “resetting” the agent’s previously learned values, such that the agents were able to unlearn the previously crystallized action preferences faster, and thus acquired the reverse pattern faster than the control group.

**Figure 8.**
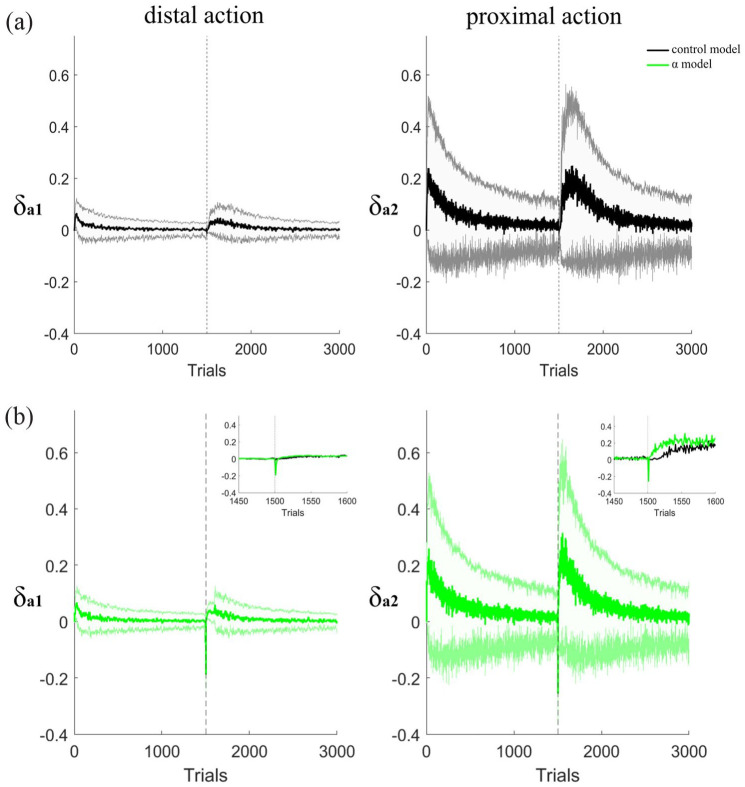
Effects of modifying the state learning rate α on the RPE. Changes in RPE in the distal and proximal positions through the first and second phase (indicated by the dotted line) in the simulations of experiment 1 for (a) control model and (b) α model. Data are displayed as mean ± standard error of the mean. Insets show a zoom in on the transition from phase 1 to phase 2 (which happens at trial 1500) displaying both the control and the α model for comparison of the differences in the RPE. These plots show the changes through the trials, not aggregated session-wise as in the other results. RPE: reward prediction error.

In this simulation, the contingencies were suddenly changed in the second phase, such that the sequence being performed so far no longer delivered a reinforcer. At this point, the agents, both in the model and in the real experiments, were expecting to end up in a high value state after performing the sequence learned, but given the change in contingencies, they were actually obtaining nothing, which should produce a negative RPE. This negative RPE was being used to change the learned values of both states and actions so that eventually the RPE was positive again. However, in the α model, because the state value learning rate was decreased, states were not being updated as fast, causing the agents to continue to expect a high state value for longer, and thus producing a more negative RPE than in the agents that did not have their state learning rate parameter reduced. Figure 8 shows the RPEs for the distal and proximal actions at each trial for the control simulations, that is, without modifications to α, in the top plots (black lines), and for the α model in the bottom plots (green lines) for both phases. At the beginning of the second phase (right after the dotted line), both control and manipulated agents had negative RPE, caused by the change in the contingencies; however, for the simulations with a smaller α, the RPE was indeed more negative in the first trials than in the control simulations (Figure 8(a) and (b)), which allowed then a larger positive RPE as shown in the inset of Figure 8(b) which shows a zoom in on the trials around the transition from phase 1 to phase 2. Since the RPE is used to update both state and action preferences, this more negative RPE was what ended up producing the larger decay in the action preferences, allowing a faster extinction process of the first learned sequence.

**Figure 9.**
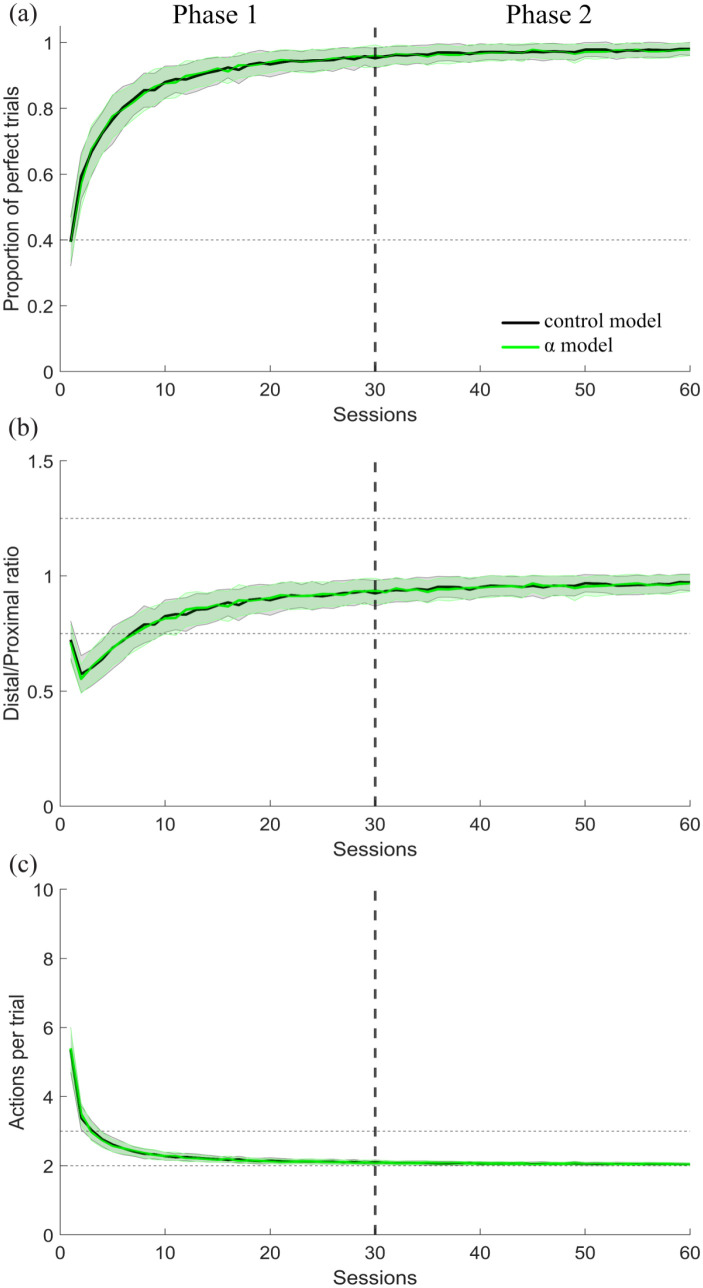
Effects of modifying the state learning rate α on the performance of a well-learned action sequence. Results for the simulations of experiment 2, in which parameter α was manipulated in the first 100 trials of the second phase for the α model in comparison to the control model are shown for (a) proportion of perfect trials, (b) distal/proximal ratio, and (c) actions per trial. Data are shown as mean ± standard error of the mean.

With this finding, we moved on to test whether this same manipulation, decreasing α, would reproduce the results from the second performance experiment. To do this, we trained another two batches of 100 simulated agents to learn action sequence LR for 30 sessions. Then, in a second phase we continued to reinforce the same sequence, but for the next 100 trials, α was set to 0.03 for one of the batches (α model), and left unaffected for the other batch of agents (control model). Figure 9 shows that the manipulation of α had no effect on the performance of a learned sequence, with no differences between the control and α agents’ performance of perfect trials (Figure 9(a)), distal/proximal ratio (Figure 9(b)) or actions performed per trial (Figure 9(c)), replicating our second experiment, in which the NK1 receptor antagonist had no effect on the stable performance of the well-learned sequence (Figure (5)). In terms of the reinforcement learning model, it makes sense that no effect was observed, since modifying the learning rates once the values of the state and actions have been already learned has little impact since there is not much left to learn, unless the contingencies changed.

Because the correlation between the model’s terms and the biological structures/functions are rather loose, we cannot reject a priori the possibility that other parameter variations could have led to the experimental effects observed. Therefore, to test that the effects we obtained in the simulations were specific to decreasing parameter α, we tested whether decreasing: (1) the action preferences learning rate, β; (2) the eligibility trace’s decay parameter, λ; and (3) the discount parameter, γ, would replicate the effects found on both behavioral experiments. For each of these hypotheses we ran another 100 simulations of the first learning experiment. We did not find that any of these alternative hypotheses to be able to correctly reproduce our experimental results (Figure S3, Supplemental Material). In summary, manipulating α was able to reproduce the counter-intuitive finding that administering an NK1 receptor antagonist led to learning a new sequence faster without affecting the stable performance of a crystallized sequence, replicating the results from both of our experiments through an effect on the RPE.

## Discussion

As behavioral sequences are learned and their performance crystallized, specific activity patterns emerge in the striatum (Jin et al., 2014; Rothwell et al., 2015), which have been suggested to contribute to the concatenation and representation of sequences as units (Jin et al., 2014; Wymbs et al., 2012). Striatal MSNs are known to interact with each other through extensive collaterals (Tepper et al., 2008; Wilson and Groves, 1980), through which they release not only fast-acting neurotransmitters such as GABA, but also more slowly acting neuromodulators such as SP, a neuropeptide with facilitatory effects on cortico-striatal synapses and striatal dopamine release (Blomeley and Bracci, 2011; Brimblecombe and Cragg, 2015; Chen et al., 2001; Graybiel, 1990). However, the role of SP in sequence learning is not fully understood. Here we show initial evidence that an NK1 receptor antagonist decreased the stable retention of a well-learned sequences, facilitating its extinction, which allowed the faster acquisition of a new sequence in a reversal learning task. Additionally, using a reinforcement learning model we propose that SP could be acting, at least in part, through the state value learning rate, modulating the size of the RPE, believed to be encoded in the dopaminergic signal.

Our proposal is consistent with electrophysiological findings and other reinforcement learning models. It has been shown that the NK1 receptor antagonist L-733,060 affects striatal dopamine concentration in rats’ striatum (Kraft et al. 2001; Noailles and Angulo, 2002). Furthermore, SP has been shown to interact with dopamine differently depending on the striatal compartment, upregulating dopamine in striosomes, while leaving it unaffected in the matrix (Brimblecombe and Cragg, 2015). Moreover, the striatum’s striosomal compartments have been suggested to encode state values, while matrix compartments, action values (Amemori et al., 2011; Doya, 2002; Shivkumar et al., 2017). Thus, injecting the NK1 receptor antagonist could have affected dopamine release only in striosomes, thereby only modifying the effects of the RPE on state values. This supports our hypothesis of mapping the effects of the NK1 receptor antagonist to decreasing the learning rate of the state values in our model of sequence learning. Our model suggested that decreasing the state values’ learning rate produces a more negative RPE, leading to a “reset” of the action values, which is a plausible explanation as to why we observed facilitated extinction in the behavioral experiments. Nonetheless, there is a possibility that the effect of the NK1 receptor antagonist was not solely based on NK1 receptors and dopamine, since there is evidence that some NK-1 antagonists display non-specific binding, in particular to L-type calcium channels (CP-96,345: Guard et al. 1993; RP-67580: Rupniak et al. 1993; Dudley et al. 2013, Weir et al. 2014) and that species differences in terms of binding exist, with lower affinity in rats than in gerbils and humans (Duffy et al., 2002; Griffante et al., 2006). However, to our knowledge the binding of L-733,060 to L-type channels has not been described yet.

Our model suggests that striosomes play an important role in the calculation of the RPE. Brown et al. (1999) more than 20 years ago, already suggested the negative RPE encoded by a decrease in dopamine is dependent on the projections from striosomes to the substantia nigra pars compacta. Thus, the idea that striosomes are fundamental for the RPE has been around for a while (Joel et al., 2002). More recent models, like those of Amemori et al. (2011) and Shivkumar et al. (2017), have further suggested that striosomes have representations of state values, whereas the matrix, of action values. Our results support these proposals, but we add the particular interaction of SP with dopamine in striosomes reported by Brimblecombe and Cragg (2015), successfully replicating the behavioral results from both of our experiments.

It is possible that our experimental result could have been due to differences in learning, motivation, or attention caused by the injection of the NK1 receptor antagonist; however, our data shows that this is unlikely. First, rats injected with saline and the NK1 receptor antagonist had similar pre-drug learning curves, suggesting that there were no baseline differences in their learning abilities. Furthermore, the NK1 receptor antagonist did not affect the inter-response times of the sequences, meaning that it did not affect the performance speed, suggesting that general motor abilities were not impaired. Likewise, the fact that we did not find any differences in the time between lever presses and the total time needed to perform the sessions up to completion, suggests that the NK1 receptor antagonist administration did not cause the rats to become inattentive either at a small scale (in between the presses) or a large scale (the overall duration of the sessions). Finally, the faster extinction of the first sequence in the first experiment was not the result of an overall decrease in motor output given that there was no decrease in the performance of the new sequence being learned. This also indicates that the differences observed were not due to modifications in overall motivation, given that both groups were equally motivated to obtain the reinforcers, obtaining them at similar rates. Finally, injecting the NK1 receptor antagonist in a reversal learning task like our first experiment, allowed us to control for novelty effects, given that the rats were already familiarized with the task and chambers.

Nevertheless, there are several other mechanisms that could have played a part in our results. Besides the effect of SP on dopamine, SP is also known to have a facilitatory effect on cortico-striatal synapses, which the model of Buxton et al. (2017) has recently proposed to be a key mediator of action chunking, and thus another mechanism by which NK1 receptor antagonism could have affected sequence learning. Additionally, NK1 receptors are also found on cholinergic interneurons in the striatum (Chen et al., 2001), and SP is known to increase the response of these interneurons and thus the release of acetylcholine in the striatum in freely moving rats (Anderson et al., 1993; Aosaki and Kawaguchi, 1996). This is another important possible mechanism by which NK1 receptor antagonism could have affected sequence learning since the activation of striatal cholinergic interneurons has been associated with habit substitution (Aoki et al., 2018). Given that we did a systemic intervention, other brain structures could have been involved. For example, some of the activity patterns that are believed to represent a learned sequence as a unit in the striatum, such as the start/stop signals, are known to have parallels in other areas, such as prefrontal cortex (Fujii and Graybiel, 2003; Smith and Graybiel, 2016). Finally, it is worth noting that NK1 receptors have been extensively linked to attentional processes, and are believed to play a key role in ADHD (Yan et al., 2010; Weir et al., 2014; Porter et al., 2015; Porter et al., 2016); thus, it is possible that by antagonizing NK1 receptors, we affected attentional processes in a way that promoted the learning of a new sequence faster.

Although neuropeptides have been neglected in the study of sequence learning, recent findings have implicated SP in action sequencing (Buxton et al., 2017; Favila et al., 2021). Our results suggest that administering an NK1 receptor antagonist led to a faster disintegration of a well-learned sequence. With these results and our RL model we propose that SP could be involved in consolidating and maintaining the stability of the representation of an action sequence through an interaction with the dopaminergic RPE. Stability is just as important as plasticity for learning new information, playing a role in preventing constant forgetting (Mermillod et al., 2013). Our findings could have implications for breaking hard-wired habits, such as the behavioral patterns related to the retrieval and use of drugs in addictions, which become “super habits” and thus very difficult to break (Graybiel, 2008).

## Supplemental Material

sj-docx-1-jop-10.1177_02698811231161582 – Supplemental material for The NK1 antagonist L-733,060 facilitates sequence learning Click here for additional data file. Supplemental material, sj-docx-1-jop-10.1177_02698811231161582 for The NK1 antagonist L-733,060 facilitates sequence learning by Natalia Favila, Kevin Gurney and Paul G. Overton in Journal of Psychopharmacology
