# Supplementary material for: The NK1 antagonist L-733,060 facilitates sequence learning
Source: J Psychopharmacol. 2023 Mar 29;37(6):610–26. doi: 10.1177/02698811231161582 (PMC10291388; doi:10.1177/02698811231161582)
Supplement: sj-docx-1-jop-10.1177_02698811231161582 – Supplemental material for The NK1 antagonist L-733,060 facilitates sequence learning [file sj-docx-1-jop-10.1177_02698811231161582.docx]

**Supplementary materials**

**
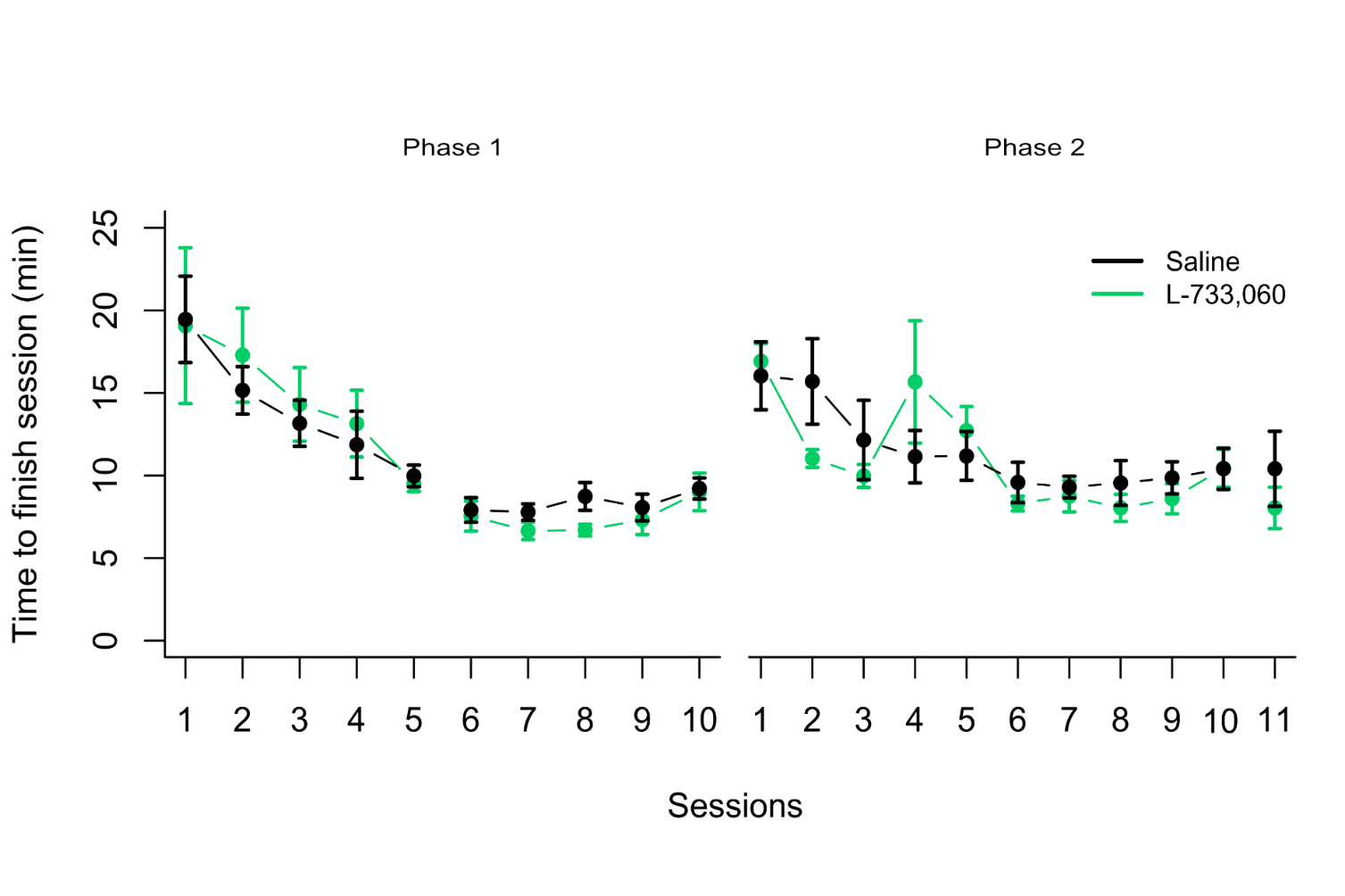
**

**Figure S1.** Total time needed to finish the sequence learning task sessions. There was no significant difference between the two groups in the second phase when the NK1 receptor antagonist was administered (F(1,9) = 0.063, p = 0.807), meaning that both the animals administered with saline and the animals administered L-733,060 were able to perform the task at a similar rate, suggesting that there were no relevant periods of inattention by the rats receiving the NK1 receptor antagonist.


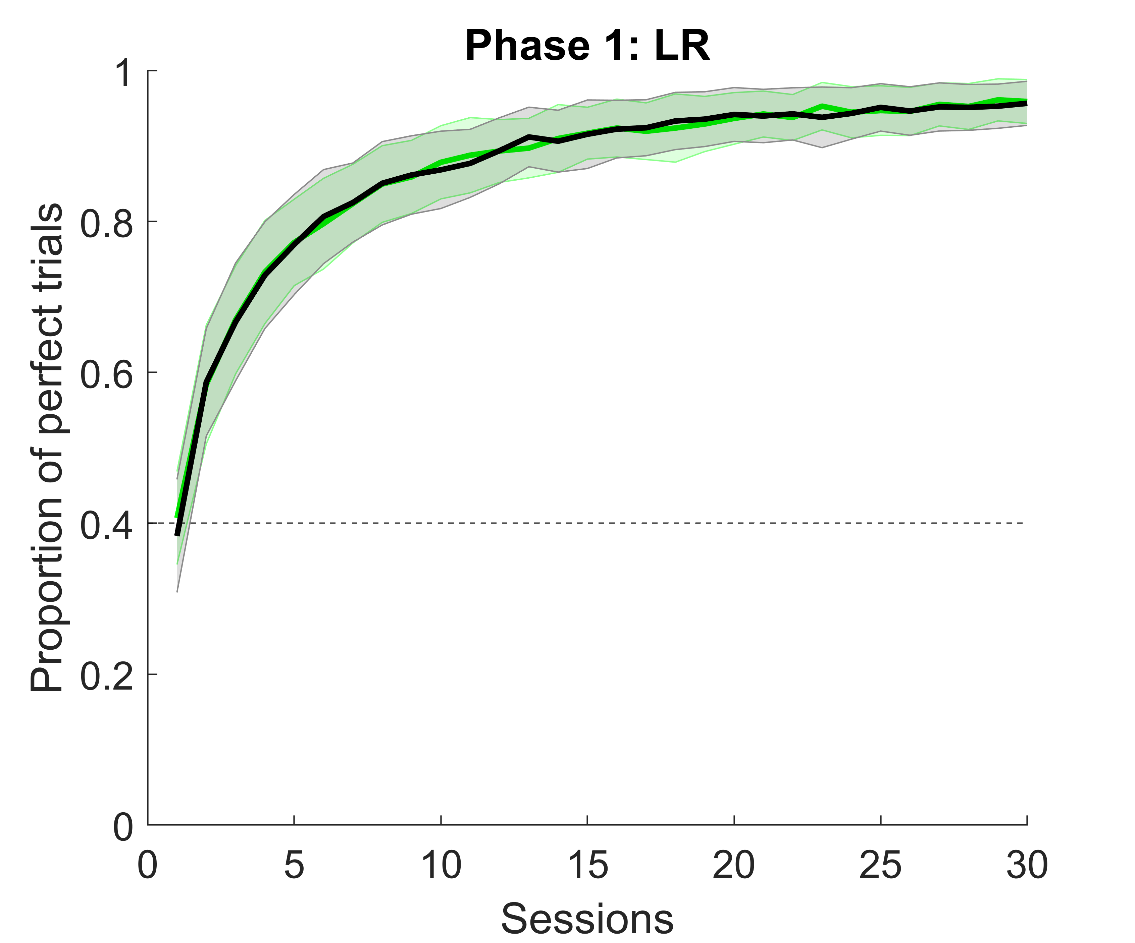


**Figure S2.** Performance of the reinforcement learning models in the first (pre-manipulation) phase. Results display the proportion of perfect trials while learning the first action sequence of the simulation of the first experiment for the agents assigned to the alpha model (green) and for the control model (black). Both batches of simulated agents learned the first sequence similarly.


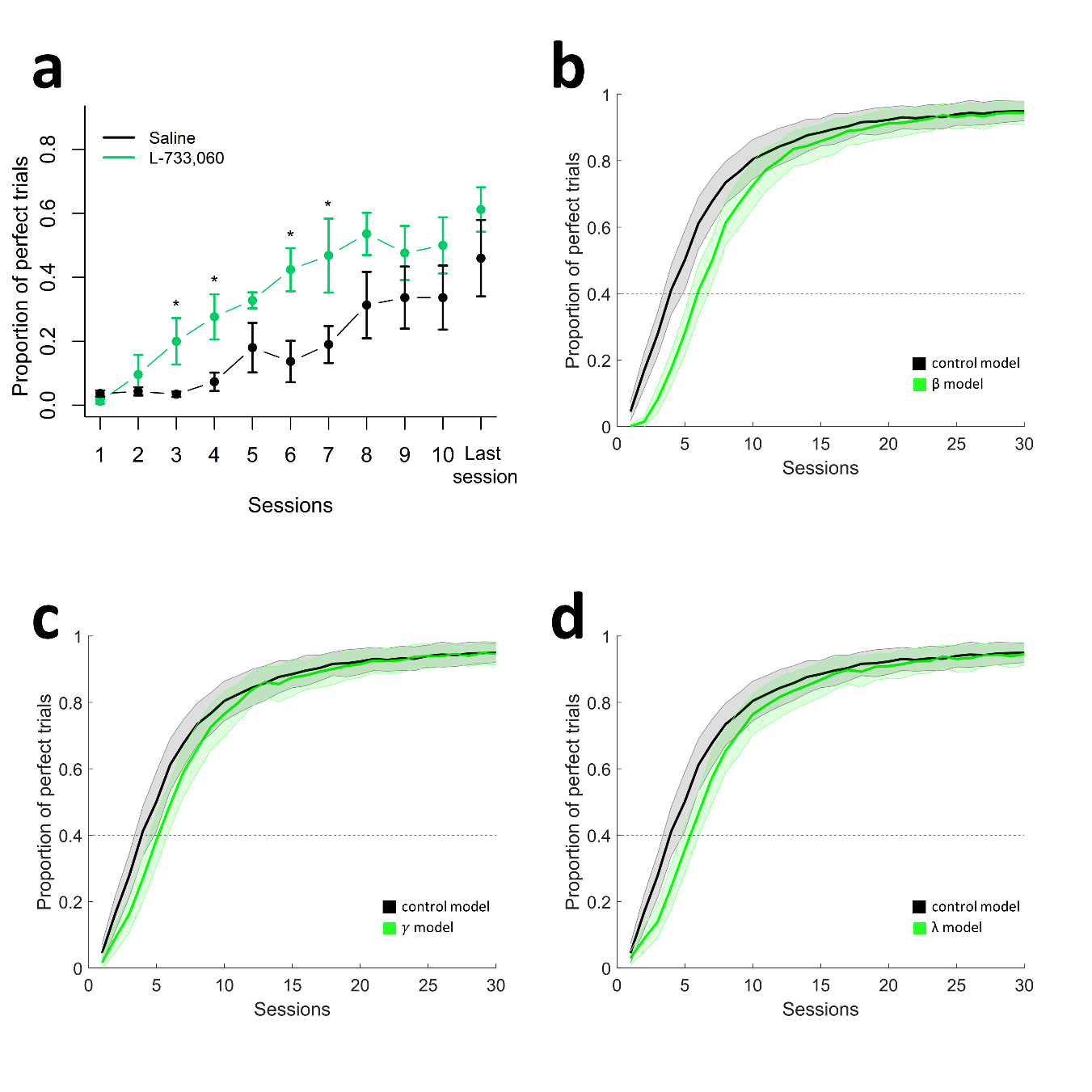
**Figure S3**. Alternative parameter manipulations - predictions for the learning experiment. Simulation 1, in which the order of the action sequence is reversed in the second phase, was used to test whether modifying other parameters of the model would create the same effect as our state learning rate hypothesis. Plots show results from the second phase for: **a**, the experimental data, where the black line is the data from the control rats and the green line the data from the rats injected with L-733,060. For the simulations the black line represents the control model with no modification of the parameters, whereas the green line represents the predictions made when: **b**, the action learning rate β was decreased in the simulations, **c**, the eligibility trace parameter $\lambda$ was decreased and, **d**, the discount factor $\gamma$ was decreased. None of these manipulations correctly predicted the experimental data.
